# Supplementary material for: Benchmark dataset for training machine learning models to predict the pathway involvement of metabolites
Source: bioRxiv. 2023 Oct 9:2023.10.03.560715. Preprint. [Version 2] doi: 10.1101/2023.10.03.560715 (PMC10592640; doi:10.1101/2023.10.03.560715)
Supplement: Supplement 1 [file media-1.pdf]

Table S1 – Parameters for configuring the atom coloring method.

Method Parameter	Parameter Description	Value We Chose	Reason For Value
r_groups	If true, add R groups in the coloring.	True	Enabled us to replace ‘R’ symbols with ‘C’ (most R-groups are bonded to the rest of the molecule beginning with a carbon) and thus include more atom color detail.
bond_stereo	If true, add bond stereo detail when constructing colors.	True	Added stereochemistry detail to atom colors which is relevant to predicting pathway involvement and more precisely distinguishes compounds.
atom_stereo	If true, add atom stereo detail when constructing colors.	True	Added stereochemistry detail to atom colors which is relevant to predicting pathway involvement and more precisely distinguishes compounds.
resonance	If true, ignore the difference between double and single bonds.	False	Resonance set to True treats all bonds as single bonds whereas setting it to False distinguishes bond order.
isotope_resolved	If true, add isotope detail when constructing colors.	False	Isotope specification of atoms does not uniquely identify a compound since identical compounds can contain atoms with different isotopes.

charge	If true, add charge detail when constructing colors.	False	Atom charge does not uniquely identify a compound since the only difference is electron content rather than elemental identity.
backbone	If true, ignore bond types in the coloring.	False	Added bond-type detail to atom colors which is relevant to predicting pathway involvement and more precisely distinguishes compounds.

Figure S1 – Distribution of mean minus median differences of feature importance scores for each pathway category in the full dataset trained on the XGBoost model

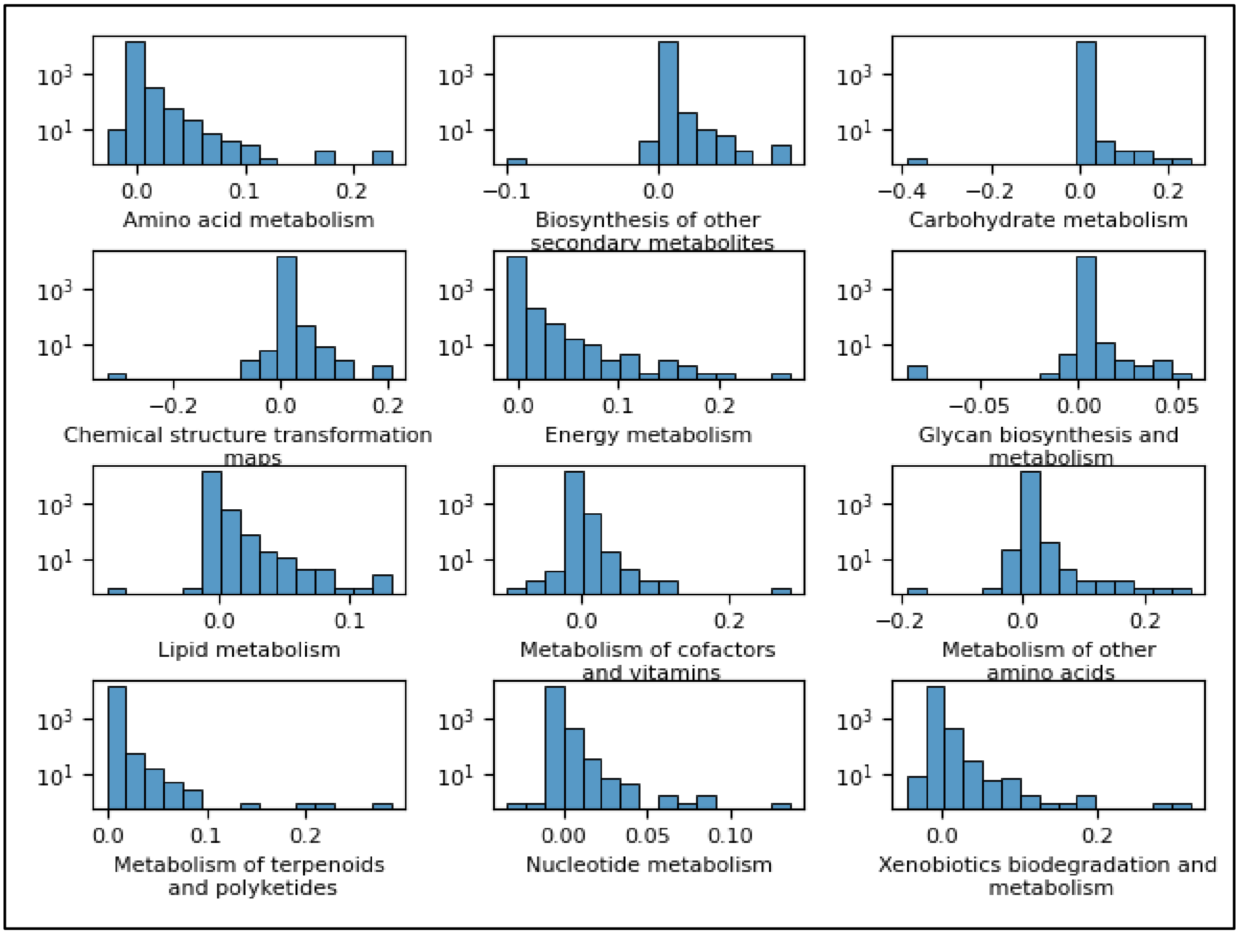

Table S4 – Pathway category proportions in each dataset.

Dataset	Pathway category	Proportion
Full	Amino acid metabolism	0.1075
	Biosynthesis of other secondary metabolites	0.2615

	Carbohydrate metabolism	0.0904
	Chemical structure transformation maps	0.0769
	Energy metabolism	0.0304
	Glycan biosynthesis and metabolism	0.0570
	Lipid metabolism	0.1191
	Metabolism of cofactors and vitamins	0.0966
	Metabolism of other amino acids	0.0482
	Metabolism of terpenoids and polyketides	0.1923
	Nucleotide metabolism	0.0297
	Xenobiotics biodegradation and metabolism	0.1652
Non-ambiguous	Amino acid metabolism	0.1109
	Biosynthesis of other secondary metabolites	0.2769
	Carbohydrate metabolism	0.0876
	Chemical structure transformation maps	0.0811
	Energy metabolism	0.0308
	Glycan biosynthesis and metabolism	0.0388
	Lipid metabolism	0.1079
	Metabolism of cofactors and vitamins	0.0976
	Metabolism of other amino acids	0.0480
	Metabolism of terpenoids and polyketides	0.1942
	Nucleotide metabolism	0.0310
	Xenobiotics biodegradation and metabolism	0.1752
Unfiltered	Amino acid metabolism	0.1117
	Biosynthesis of other secondary metabolites	0.2517
	Carbohydrate metabolism	0.0965
	Chemical structure transformation maps	0.0725
	Energy metabolism	0.0397
	Glycan biosynthesis and metabolism	0.0578
	Lipid metabolism	0.1265
	Metabolism of cofactors and vitamins	0.1055
	Metabolism of other amino acids	0.0567
	Metabolism of terpenoids and polyketides	0.1851
	Nucleotide metabolism	0.0317
	Xenobiotics biodegradation and metabolism	0.1695

Table S7 – Valid score counts less than 300.

Model	Dataset	Test Set	Pathway Category	Metric	Valid Score Count
Multi-layer Perceptron	Full	Ambiguous	Chemical structure transformation maps	Recall	229
			Nucleotide metabolism	F1 Score	219

				Recall	181
				Precision	127
			Xenobiotics biodegradation and metabolism	F1 Score	264
				Recall	215
				Precision	99
Random Forest	Full	Ambiguous	Biosynthesis of other secondary metabolites	Precision	159
			Chemical structure transformation maps	Recall	229
			Energy metabolism	Precision	69
			Metabolism of other amino acids	Precision	184
			Nucleotide metabolism	F1 Score	194
				Recall	181
				Precision	81
			Xenobiotics biodegradation and metabolism	F1 Score	270
				Recall	215
				Precision	142
	Non-ambiguous	Ambiguous	Metabolism of other amino acids	Precision	200
			Nucleotide metabolism	Precision	1
XGBoost	Full	Ambiguous	Chemical structure transformation maps	Recall	229
				Precision	149
			Energy metabolism	Precision	269
			Nucleotide metabolism	F1 Score	190
				Recall	181
				Precision	182
			Xenobiotics biodegradation and metabolism	Recall	215
				Precision	240

Figure S2 – MCC By test set for each pathway category for the XGBoost model trained on the full dataset.

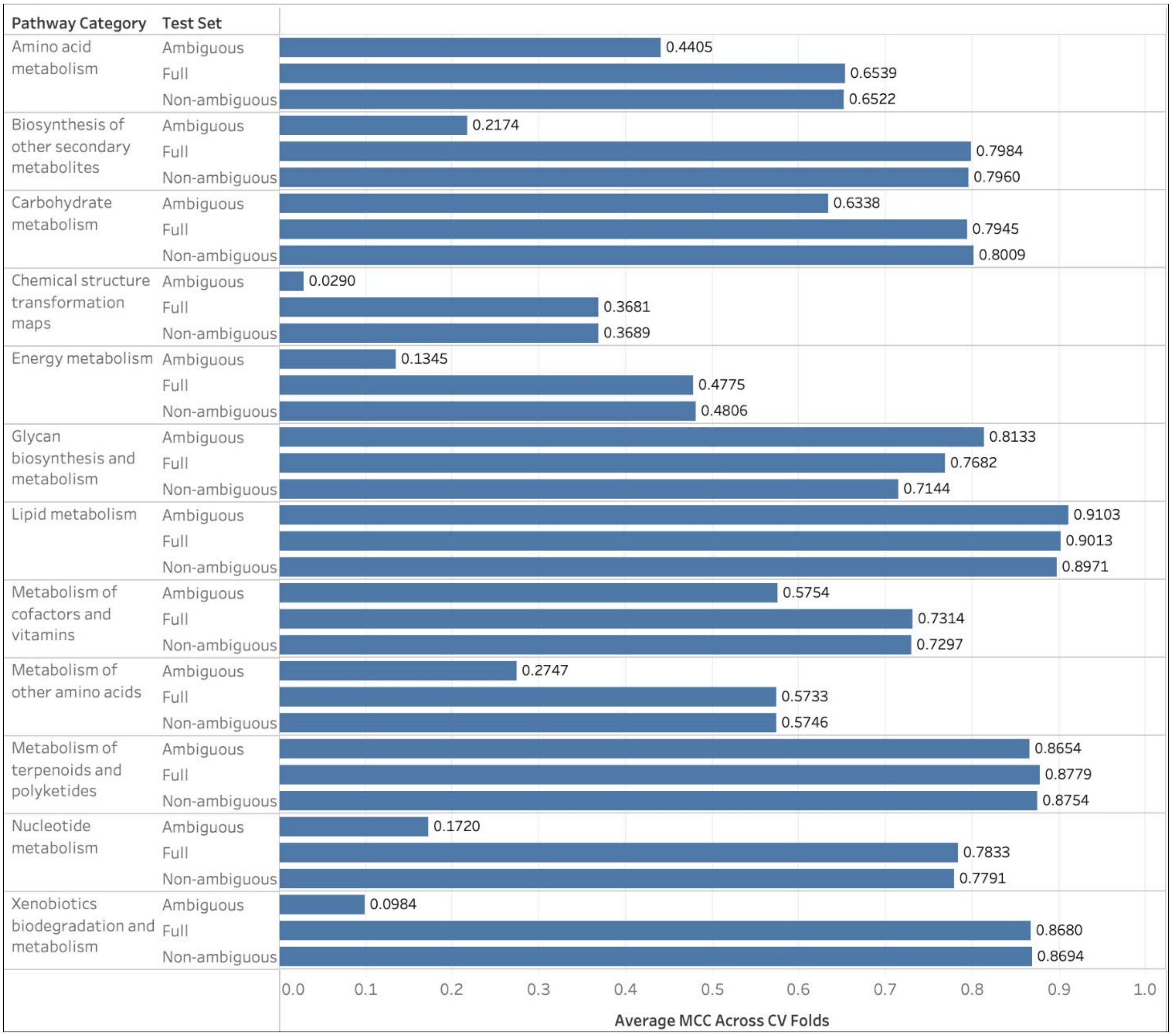

Figure S3 – Upset plot showing overlap between pathway categories of their top 50 most important features.

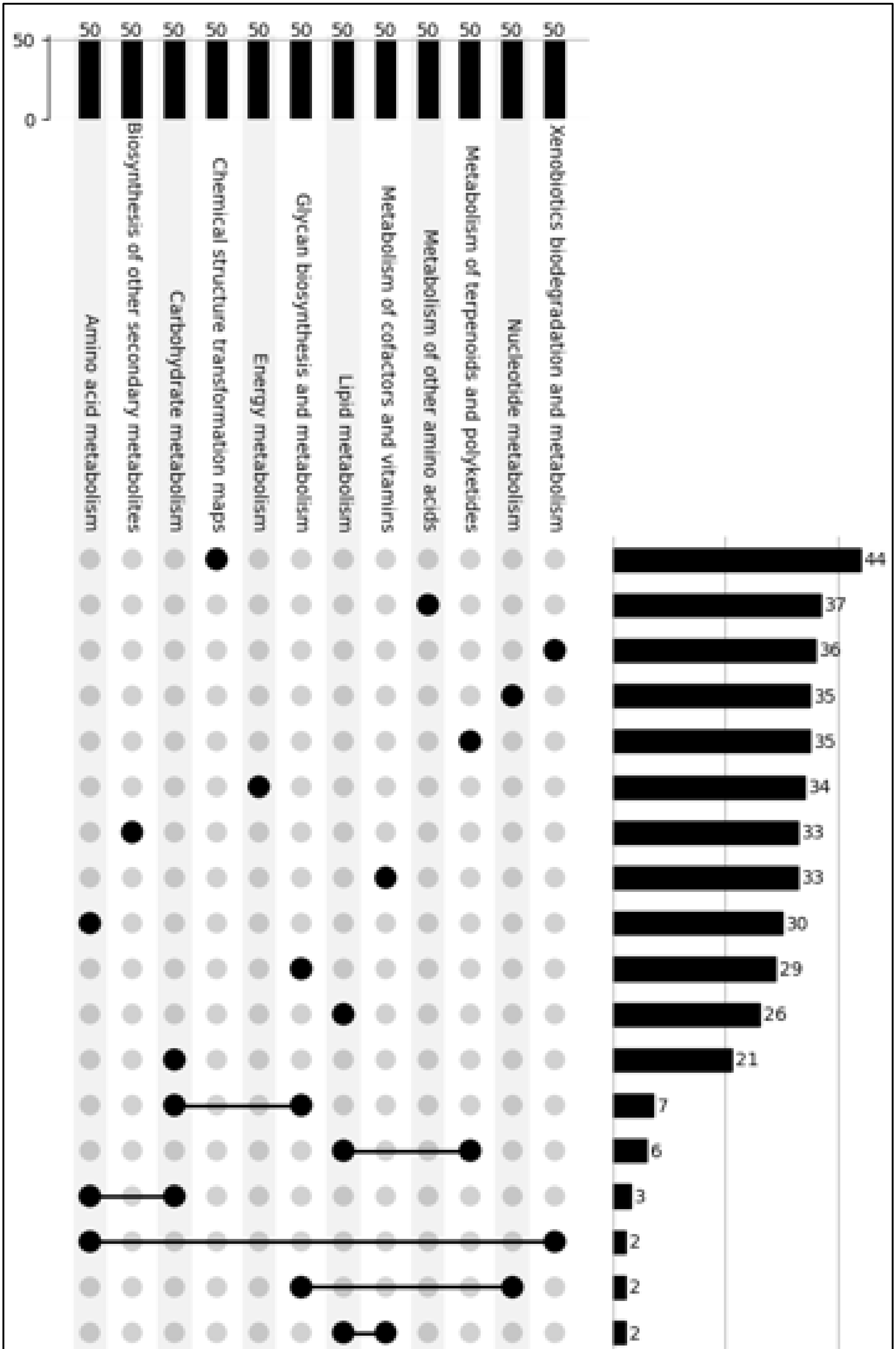

50
0
50
Amino acid metabolism
Biosynthesis of other secondary metabolites
Carbohydrate metabolism
Chemical structure transformation maps
Energy metabolism
Glycan biosynthesis and metabolism
Lipid metabolism
Metabolism of cofactors and vitamins
Metabolism of other amino acids
Metabolism of terpenoids and polyketides
Nucleotide metabolism
Xenobiotics biodegradation and metabolism
44
37
36
35
35
34
33
33
30
29
26
21
7
6
3
2
2
2
